# Analog Signal Summation for Reinforcement Learning via Simultaneous Light–Voltage Modulation in a Synaptic Device

**DOI:** 10.1002/advs.202521293

**Published:** 2025-12-12

**Authors:** Dong Gue Roe, Sungjoon Cheon, Seongil Im, Sinil Choi, Meeree Kim, Subeen Kim, Youngjae Yoo, Jeong Won Kim, Hyunsu Ju, Sohee Jeong, Jeong Ho Cho

**Affiliations:** ^1^ Department of Chemical and Biomolecular Engineering Yonsei University Seoul 03722 Republic of Korea; ^2^ Department of Electrical and Systems Engineering University of Pennsylvania Philadelphia PA 19104 USA; ^3^ Department of Energy Science (DOES) Center for Artificial Atoms Sungkyunkwan University (SKKU) Suwon 16419 Republic of Korea; ^4^ SKKU Institute of Energy Science and Technology (SIEST) Suwon 16419 Republic of Korea; ^5^ Post‐Silicon Semiconductor Institute Korea Institute of Science and Technology Seoul 02792 Republic of Korea; ^6^ GIST InnoCORE AI‐Nano Convergence Institute for Early Detection of Neurodegenerative Diseases Gwanjgu Institute of Science and Technology Gwangju 61005 Republic of Korea; ^7^ Department of Energy Science (DOES) Sungkyunkwan University (SKKU) Suwon 16419 Republic of Korea; ^8^ Center for Artificial Atoms Sungkyunkwan University (SKKU) Suwon Gyeonggi‐do 16419 South Korea; ^9^ Korea Research Institute of Standards and Science (KRISS) Daejeon 34113 South Korea; ^10^ University of Science and Technology (UST) Daejeon 34113 South Korea; ^11^ Department of Advanced Materials Engineering Chung‐Ang University Anseong‐si 17546 Republic of Korea

**Keywords:** artificial intelligence, reinforcement learning, single‐device computation, synaptic transistor, quantum dot

## Abstract

Major breakthroughs in artificial intelligence software have led to significant transformations across various aspects of life. However, hardware development has lagged behind, primarily due to the inherent constraints of the von Neumann architecture. Although neuromorphic devices that utilize biomimetic parallel and analog computations have emerged, they still face limitations in reducing computational load. Therefore, this study proposes a light‐voltage dual‐modulating synaptic transistor that can significantly lower computational load through device‐level computing. This is realized using a hybrid structure of indium‐gallium‐zinc‐oxide and InAs quantum dots, which enable two distinct memory effects ‒ one induced by light and the other by voltage ‒ within a single device. These dual‐modulation capabilities are leveraged to demonstrate traffic signal optimization using a Dueling Deep Q‐Network, achieving computation performance comparable to ideal software conditions. These findings highlight the potential of the fabricated device for realizing computing systems that require high energy efficiency and computational density.

## Graphical abstract

To overcome limitations of conventional AI hardware, a light‐voltage dual‐modulating synaptic (LVDS) transistor using an IGZO/InAs quantum dot hybrid structure is proposed. LVDS transistor enables analog summation for Dueling Deep Q‐Networks by independently modulating memory via optical and electrical stimuli. This device‐level computing potentially reduces computational loads, energy, and complexity by eliminating peripheral circuits.

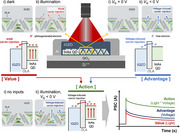

## 1 Introduction

Significant breakthroughs in software engineering and artificial intelligence (AI) have fundamentally transformed both industrial processes and everyday applications.^[^ ^1^, ^2^ ^]^ AI systems have substantially enhanced conventional computing tasks, particularly those involving repetitive pattern recognition and data processing. Moreover, AI capabilities have expanded into and dominated domains that were traditionally considered exclusive to human intelligence, such as creative content generation and complex decision‐making, demonstrating remarkable versatility.^[^ ^3^, ^4^, ^5^ ^]^ Despite these remarkable advancements in AI algorithms and software frameworks, the development of supporting hardware architectures has faced significant challenges.^[^ ^6^ ^]^ This disparity primarily stems from the inherent limitations of conventional computing architectures, particularly in terms of energy efficiency and computational density. Current hardware implementations considerably struggle to process the massively parallel operations required by modern artificial neural networks, resulting in critical bottlenecks in power consumption and computational throughput.^[^ ^7^, ^8^, ^9^, ^10^, ^11^ ^]^

Although neuromorphic devices^[^ ^12^, ^13^, ^14^, ^15^, ^16^, ^17^, ^18^, ^19^ ^]^ have demonstrated promise by incorporating parallel and analog signal processing capabilities analogous to those of biological neural systems,^[^ ^20^ ^]^ their practical realization remains significantly challenging. Existing single‐input neuromorphic architectures^[^ ^19^, ^21^, ^22^, ^23^, ^24^, ^25^ ^]^ encounter fundamental limitations in executing sophisticated neural network operations that require simultaneous processing of multiple weight matrices. This limitation becomes particularly critical in advanced neural network implementations, where parallel weight operations are essential for efficient computation. It highlights the necessity for novel device architectures that inherently support multi‐weight processing, thereby reducing overall system complexity and computational overhead.

Herein, we propose a fundamental approach to reducing AI computational loads by performing AI computations at the device level. Specifically, we demonstrate this approach using a light‐voltage dual‐modulating synaptic (LVDS) transistor fabricated with a hybrid layer of InAs quantum dots (QDs) and indium‐gallium‐zinc‐oxide (IGZO). The LVDS transistor exhibits a memory effect in response to both light and voltage signals because the oleylamine (OLA) ligands attached to the InAs QDs serve as charge‐blocking layers. By leveraging these properties, two independent input signals can be processed using a single device, eliminating the need for separate computational units and significantly reducing system complexity and computational load while ensuring linear calculation performance. To validate the effect of the device‐level analog computations of the LVDS transistor, we performed traffic signal optimization tasks using a Dueling Deep Q‐Network (DDQN) algorithm.^[^ ^26^ ^]^ Light stimuli modulate the “Value” weights (*W*_v_), while electrical stimuli control the “Advantage” weights (*W*_a_). This cooperative conductance modulation inherently realizes the final Q‐value weights (*W*_q_), enabling complex arithmetic operations at the single‐device level. The direct summation process demonstrated by the DIST and its implementation in DDQN algorithms clearly highlight the advantages of simultaneously utilizing dual input channels, extending beyond the scope of previously reported optoelectronic synaptic devices. Conventional optoelectronic synaptic devices have mainly focused on computations based on light‐induced conductance changes.^[^ ^27^, ^28^, ^29^, ^30^ ^]^ Although a few studies have explored simultaneous light and voltage stimulation, they merely demonstrated simple signal amplification or enhanced retention rather than true computational functionality.^[^ ^31^, ^32^, ^33^, ^34^, ^35^, ^36^ ^]^ The demonstrated efficient neural network implementation, along with the intrinsic advantages of our dual‐modulation approach, underscores its potential for next‐generation computing systems requiring energy efficiency, high computational density, and real‐time processing capabilities.

## 2 Results

### 2.1 Concept of LVDS Transistor‐Based AI Computation using the DDQN Algorithm

**Figure 1:**
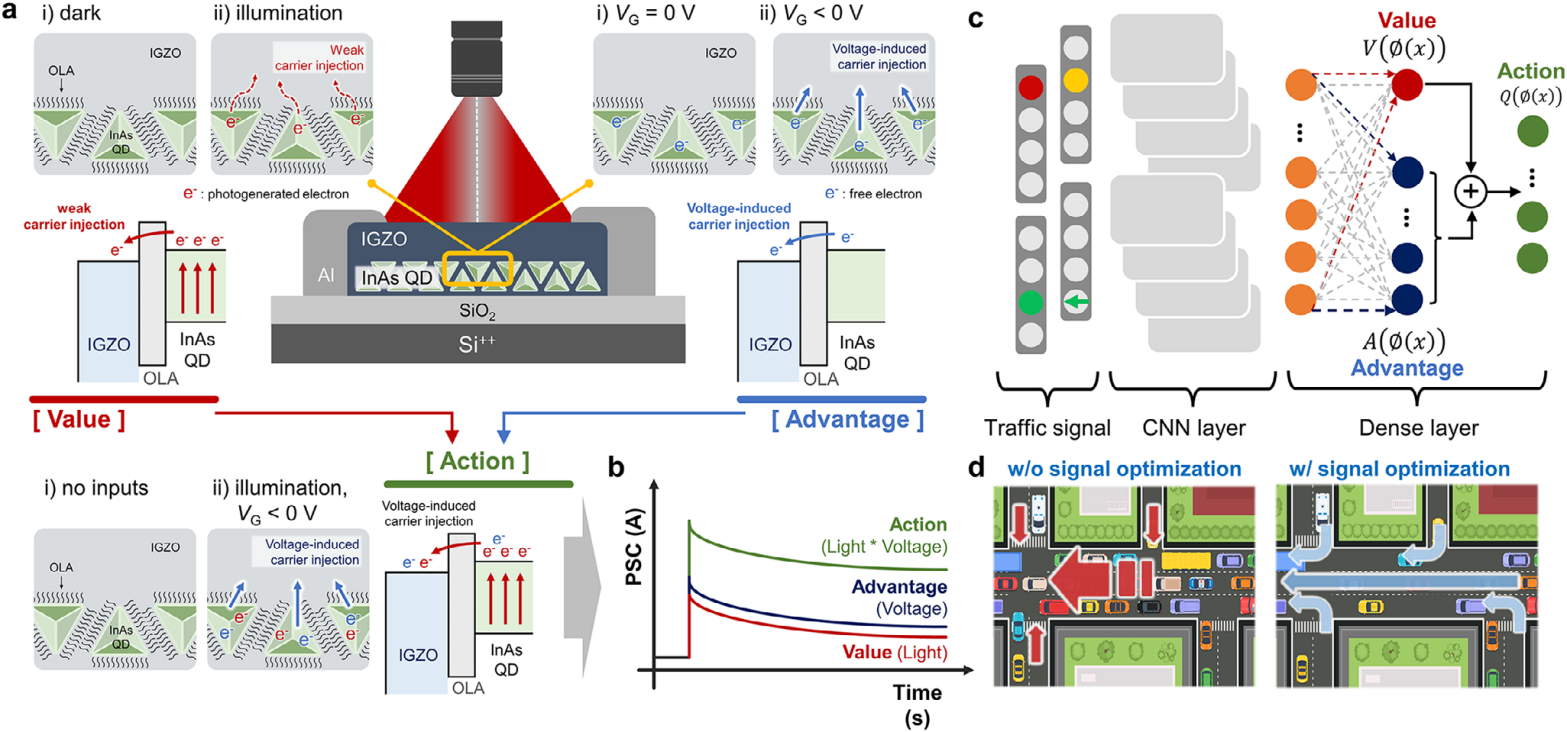
Concept of LVDS transistor‐based AI computation using the DDQN algorithm. a) Schematic diagrams illustrating the dual‐memory effect mechanism of the LVDS transistor. b) Schematic of the device‐level signal processing capabilities of the LVDS transistor. c) Schematic diagram of the DDQN algorithm for traffic signal optimization. d) Comparison of the traffic conditions before and after optimization reveals improvements in the traffic flow efficiency of the DDQN‐based approach.

**Figure** **1**a,b present schematic diagrams illustrating the dual‐memory effect mechanism of the LVDS transistor and its corresponding device‐level signal processing capabilities. The LVDS transistor was fabricated using a hybrid structure consisting of InAs QDs and IGZO (Figure S1, Supporting Information). In contrast to the conventional approach in which QD ligands are often shortened to facilitate charge transport, we deliberately retained the long OLA ligands in the InAs QDs. These ligands serve as a weak charge‐blocking layer, thereby enabling the LVDS transistor to exhibit two distinct types of memory effects: one induced by light and the other by voltage, which correspond to the “Value” and “Advantage” of the DDQN, respectively. The light‐induced memory effect arises from the limited injection of photogenerated carriers from the InAs QDs to the IGZO. Charge transfer between the InAs QDs and IGZO occurs readily due to the near alignment of their conduction bands and Fermi levels. However, the direction of charge transfer is effectively restricted from the QDs to the IGZO, as IGZO does not absorb light in the 1060 nm wavelength range used in this study, whereas InAs QDs can absorb light at this wavelength. Nonetheless, the presence of the long‐chain OLA ligands limits the number of injected carriers, resulting in a relatively weak memory effect. Conversely, the voltage‐induced memory effect primarily arises from the electric field applied between the InAs QDs and the IGZO. This external field promotes charge injection from the InAs QDs to the IGZO, allowing charge carriers to be injected readily. However, the number of carriers present in the InAs QDs under this condition is smaller than that generated by photonic excitation, resulting in a memory effect similar in magnitude to that induced by light. When light and voltage are applied simultaneously, the memory effect is amplified because the photogenerated carriers and the external electric field work together to facilitate charge injection from the InAs QDs to the IGZO. These light‐induced, voltage‐induced, and amplified memory effects correspond to the “Value,” “Advantage,” and “Action” in the DDQN, respectively (Figure 1b).

Figure 1c shows a schematic diagram of the DDQN algorithm, illustrating its approach to traffic signal optimization. In this architecture, traffic signal data are first processed through convolutional neural networks to extract features such as traffic density, vehicle distribution, and flow patterns. These abstracted features are subsequently fed into the DDQN framework, which uniquely decomposes Q‐value estimation into two parallel streams: the “Value” stream and the “Advantage” stream, which correspond to the optical and electrical modulation capabilities of the device, respectively. The Value stream evaluates the overall quality of the current traffic state, while the Advantage stream assesses the relative benefits of different possible actions. As illustrated in Figure 1d, a comparison of the traffic conditions before and after optimization revealed that our DDQN‐based approach demonstrated improved traffic flow efficiency.^[^ ^37^, ^38^ ^]^ This successful demonstration of traffic signal optimization validates the effectiveness of our dual‐modulation device architecture for real‐world applications requiring decision‐making capabilities.

### 2.2 Characterization of the InAs QDs and IGZO

**Figure 2:**
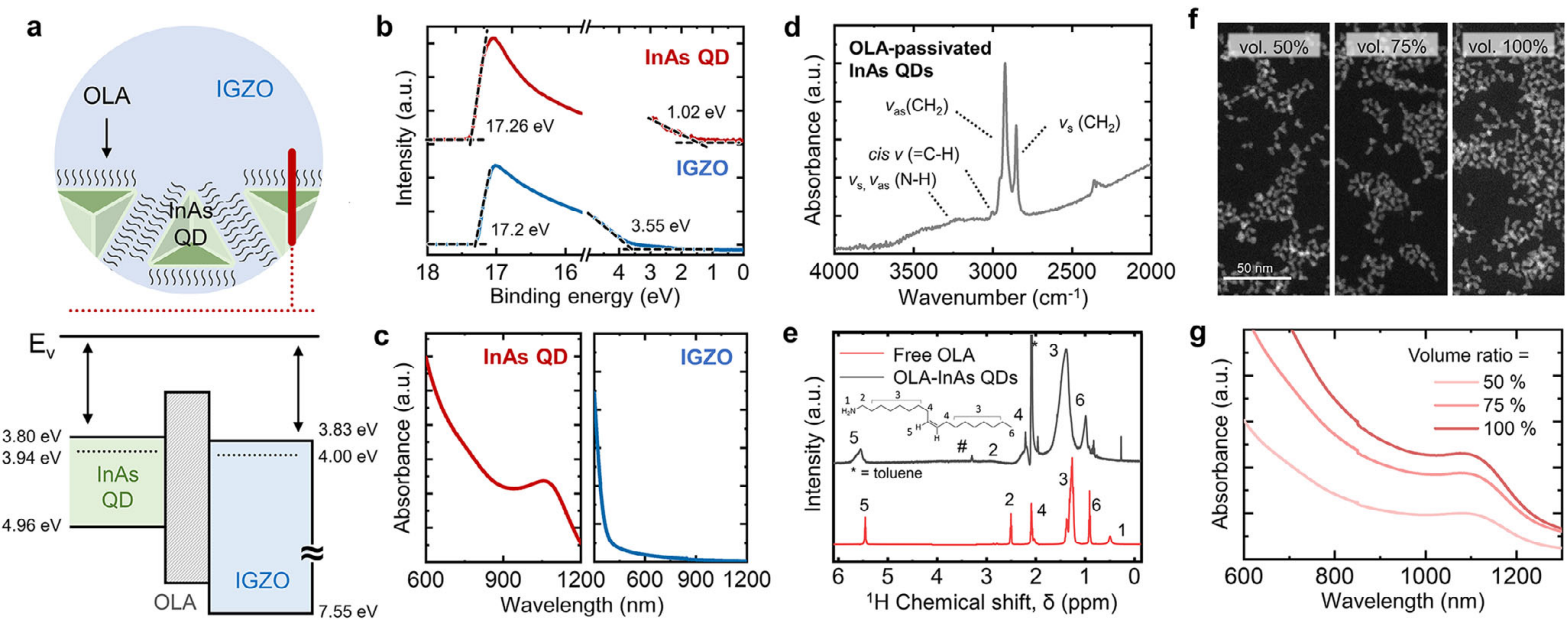
Characterization of the InAs QDs and IGZO. a) Schematic diagram of the hybrid layer comprising InAs QDs and IGZO and their corresponding band alignment. The energy levels of OLA are illustrated based on expected values for a non‐cojugated insulating organic molecule. b) UPS spectra of the InAs QDs and IGZO. c) UV–vis absorbance spectra of the InAs QDs and IGZO. d) FT–IR spectrum and e) 1H NMR spectrum of the InAs QDs. f) TEM images of InAs QDs deposited on a carbon‐coated Cu grid. g) UV–vis spectra of the InAs QDs at varying volume ratios.

**Figure 3:**
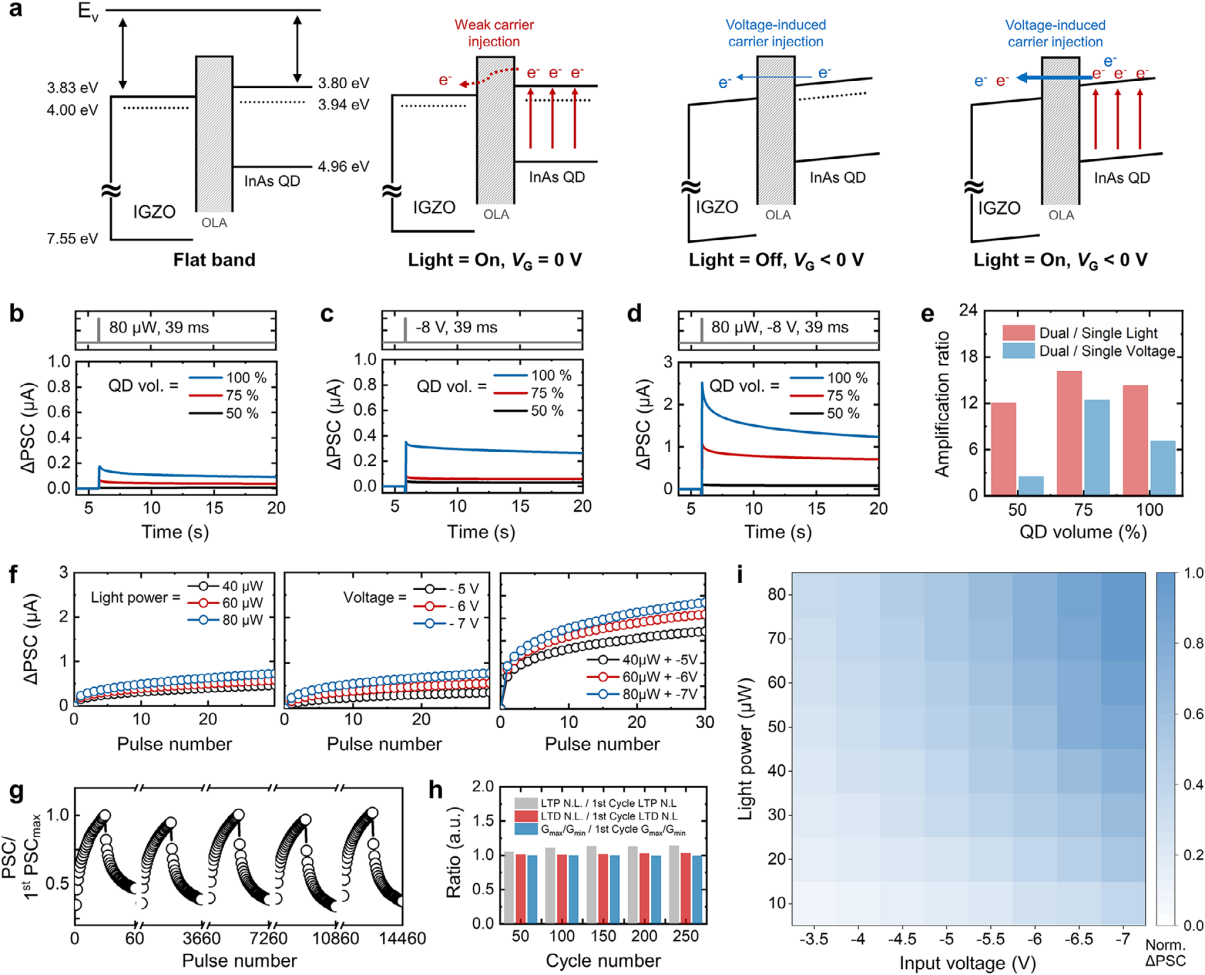
Electrical characterization of the LVDS transistor for device‐level AI computation. a) Schematic band diagram illustrating charge injection phenomena under varying input conditions. PSC change under b) a single light pulse (39 ms, 80 µW), c) a single voltage pulse (39 ms, −8 V), and d) simultaneous light and voltage pulses for various volume ratios of spin‐coated InAs QD solutions (VDS = 1 V). e) Quantitative comparison of the PSC amplification ratio under dual stimuli and a single stimulus for different volume ratios of spin‐coated InAs QD solutions. f) LTP curves of the LVDS transistor under various input conditions. g) Device stability under several consecutive pulses. h) Quantitative performance comparison under consecutive pulses. i) Combined PSC outputs under various combinations of light and voltage inputs.

**Figure** **2**a presents a schematic diagram of the hybrid layer composed of InAs QDs and IGZO and their corresponding band alignment. The OLA ligand attached to the InAs QDs blocks the interface between the QD surface and the IGZO layers (further illustrated in Figure 3). The band structures of both materials were characterized using ultraviolet photoelectron spectroscopy (UPS) and UV–vis spectrophotometry. Figure 2b shows the UPS spectra of the two materials coated on a gold substrate, with the red and blue curves representing the valence band spectra of the InAs QDs and IGZO, respectively. The work function (*Φ*) was calculated as *Φ* = 21.2 eV − *E*_SEC_ (where SEC denotes the secondary electron cutoff), and the location of the valence band maximum (*E*_VBM_) relative to the Fermi level (*E*_F_) was identified. The conduction band edge was determined using the band gap energies of the InAs QDs and IGZO, which were estimated from their absorption spectra acquired in solution and film form, respectively (Figures 2c; S2, Supporting Information). Since the InAs QDs were synthesized with OLA ligands, Fourier‐transform infrared (FT–IR) spectroscopy and ^1^H nuclear magnetic resonance (NMR) spectroscopy were conducted to confirm their presence on the QD surface. Figure 2d displays the FT–IR spectrum of the InAs QDs, with peaks at ≈2920 and ≈2850 cm^−1^ corresponding to C–H asymmetric and symmetric stretching vibrations, respectively. Characteristic OLA peaks appeared between 3300–3500 cm^−1^, corresponding to N–H stretching vibrations of the amine group, and weak alkene vibrations appeared near ≈3000 cm^−1^. These assignments are further supported by the ^1^H NMR spectrum (Figure 2e), where the alkene resonances and α‐NH_2_ proton resonances are labeled as “5” and “2,” respectively. The broadened overall signals and the deshielded α‐NH_2_ protons confirm that the OLA molecules were bound to the InAs QD surface. Direct measurement of the electronic structure of OLA was not feasible due to its liquid form and insulating nature, which led to instability and charging effects under high vacuum conditions for photoelectron spectroscopy. However, as a non‐conjugated aliphatic molecule, OLA is expected to have a wide HOMO‐LUMO energy gap (>5 eV), with energy levels far from the QD band edges. Accordingly, the energy levels in Figures 2a (and 3a) are drawn based on these expected values, as noted in the captions. Figure 2f shows the TEM images of InAs QDs deposited on a carbon‐coated Cu grid. The stock InAs QD solution (50 mg mL^−1^ in dichlorobenzene) was used at three different concentrations: undiluted (100%), and diluted to 75%, 50% of the original concentration. As the volume ratio of the spin‐coated InAs solution increased, the aerial density of the InAs QDs increased from 18.74% to 27.23% (Figure S3, Supporting Information). Moreover, UV–vis spectroscopy revealed that the absorbance increased accordingly with QD concentration (Figure 2g). Since all films were deposited on identical substrates under the same spin‐coating conditions, the variation in absorbance reflects differences in the aerial QD density of the films.

### 2.3 Electrical Characterization of the LVDS Transistor for Device‐Level AI Computation

**Figure** **3**a shows the schematic band diagram illustrating the charge injection phenomena under varying input conditions. According to the material characterization results presented in Figure 2, the InAs QDs and IGZO exhibited similar conduction bands and Fermi levels, which facilitate charge transfer between the two layers. However, the presence of the OLA ligand, acting as a charge‐blocking layer,^[^ ^39^, ^40^, ^41^ ^]^ limits charge transfer in the absence of external stimuli due to its long molecular structure. For example, when only light is applied to the LVDS transistor, the number of carriers injected into the IGZO layer remains limited despite the large number of photogenerated carriers present in the conduction band of the InAs QDs. This occurs because the OLA ligands block injection, resulting in weak carrier injection. Conversely, when only a negative voltage is applied to the gate electrode of the LVDS transistor, the induced electric field between the InAs QDs and IGZO facilitates the injection of carriers into the IGZO layer. However, under these conditions, only a small number of carriers exist in the conduction band of the InAs QDs, resulting in a similarly low level of carrier injection. When both light and voltage stimuli are applied, carrier injection is synergistically enhanced, as photogenerated carriers in the InAs QDs can be more efficiently injected into the IGZO channel under the electric field induced by the voltage input. The charge‐blocking‐induced memory capability of the OLA‐attached InAs QDs was quantitatively analyzed by comparing the performance of LVDS transistors incorporating 3‐mercapto‐1,2‐propanediol (MPD) attached InAs QDs (Figure S4a, Supporting Information). MPD was chosen as a comparative ligand due to its significantly shorter molecular structure than OLA, as the carrier mobility depends on the length of the ligand on the NC surface, where the hopping transport occurs with alkane tunnel barriers.^[^ ^42^ ^]^ Ligand exchange between OLA and MPD was confirmed by FT–IR spectroscopy (Figure S4b, Supporting Information), showing noticeably weakened C─H stretching vibrations (at 2850 and 2920 cm^−1^) and alkene vibrations near 3000 cm^−1^, characteristic of OLA, as well as the appearance of a broad O─H stretching vibration attributed to MPD. These spectral changes indicate that OLA ligands were effectively replaced by MPD, confirming the successful surface modification of the InAs QDs. The memory characteristics difference between two ligands was evaluated by measuring the post‐synaptic current (PSC) after applying 30 consecutive potentiation pulses under three different input conditions: light, voltage, and combined light + voltage (L+V) (Figure S4c,d, Supporting Information). As expected, the LVDS transistor incorporating MPD exhibited significant memory decay under all input conditions. This observation was further supported by quantitative analysis through the extraction of long‐term retention time constants from the PSC curves (Figure S4e, Supporting Information). These results clearly indicate that the ligand plays a key role in modulating the memory effect of the LVDS transistor.

Figure 3b shows the PSC change under a single light pulse (80 µW, 39 ms) for various volume ratios of spin‐coated InAs QD solutions. Detailed information about the light source can be found in the Section S1 (Supporting Information). Similar to the trend observed in Figure 2g, the retentive PSC increased as the volume ratio increased. Figure 3c shows the PSC change under a single voltage pulse (−8 V, 39 ms). As in the light stimulus condition, the retentive PSC increased with the volume ratio, and the PSC level was comparable to that induced by light stimuli. Figures 3d and S5 (Supporting Information) show the PSC change under simultaneous application of light and voltage pulses. Here, the drain voltage (*V*_DS_) of the device was fixed at 1 V for all electrical measurements. The retentive PSC under these conditions was amplified compared to that under the single‐stimulus condition. Figure 3e provides a quantitative comparison of the PSC amplification ratios under dual‐stimulus and single‐stimulus conditions for different volume ratios of spin‐coated InAs QD solutions. The PSC amplification ratio was highest at a 75% volume ratio, with the ratio under dual stimuli being more than tenfold higher than that under the single‐stimulus condition. Since a high amplification ratio is advantageous for state partitioning in AI computation, the 75% volume ratio condition was chosen for subsequent experiments.

Figure 3f shows the long‐term potentiation (LTP) curves of the LVDS transistor under various input conditions. Consistent with the single‐pulse results, single light and voltage inputs resulted in comparable PSC levels, whereas dual inputs produced notably higher PSC levels (Figure S6, Supporting Information). The long‐term depression (LTD) characteristic was evaluated by varying the input voltage amplitude (Figure S7, Supporting Information). Additional LTP/LTD tests were conducted under various input conditions with different pulse widths and numbers of pulses to verify consistent weight modulation capability across different stimuli (Figures S8, Supporting Information). Figures 3g and S9 (Supporting Information) illustrate the stability of devices under a series of consecutive pulses. The nonlinearity (*NL*) of LTP/LTD and the *G*_max_/*G*_min_ ratio remained essentially unchanged throughout the application of 15000 pulses (Figure 3h). Moreover, the device‐to‐device variation of the LVDS transistor was evaluated by measuring the hysteresis windows of 25 individual devices (Figure S10, Supporting Information), which exhibited negligible variation, indicating excellent device uniformity.

To assess the AI computation capabilities of the LVDS transistor, light and voltage signals of varying magnitudes were applied to the LVDS transistor. In response, the LVDS transistor successfully produced combined PSC outputs reflecting these changes, demonstrating its ability to perform summation operations at the device level (Figure 3i). Furthermore, the resulting PSC exhibited a linear increase according to the input signals, a feature beneficial for AI computation, validating the suitability of the LVDS transistor for AI applications.

### 2.4 Implementation of the LVDS Transistor Array for a Traffic Control System

**Figure 4:**
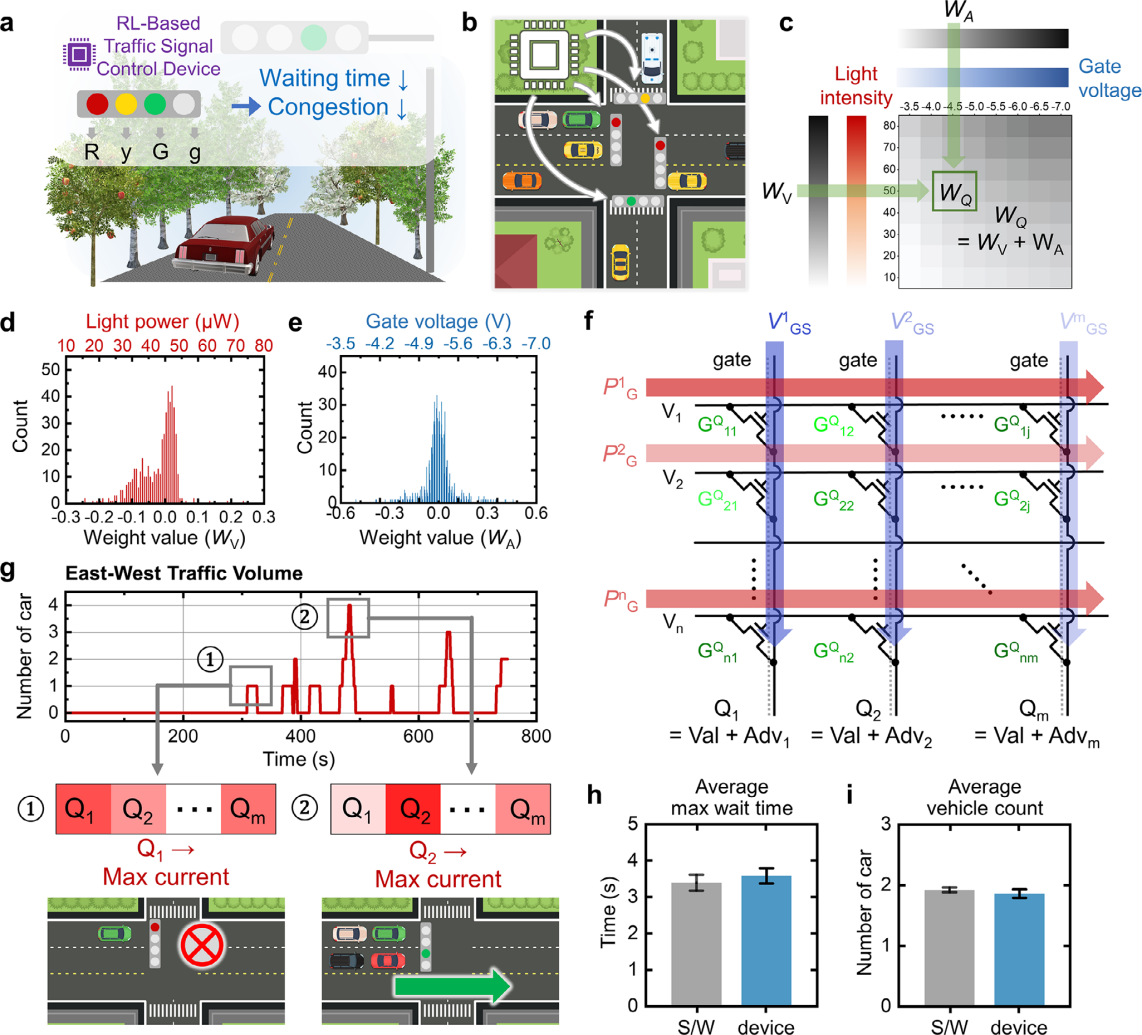
Implementation and performance evaluation of the LVDS array‐based DDQN traffic control system. a) Schematic illustration of LVDS transistor implementation for DDQN‐based traffic signal optimization. b) Architecture showing a single LVDS device controlling multiple traffic signals at an intersection. c) Device characterization showing the DDQN weight combination process. The 80×80 weight matrix used in simulation was generated by bicubic interpolation from experimentally measured 8×8 device‐level responses. DDQN weight mapping for implementation: d) mapping of value‐layer weights to light intensity and e) mapping of advantage‐layer weights to gate voltages. f) Array‐scale implementation for parallel DDQN weight processing. g) Dynamic traffic response demonstration using SUMO simulation. Performance comparison between DDQN device‐based and software simulations: h) maximum wait times and i) vehicle throughput over 100 trials.

Our LVDS transistor implementation demonstrates device‐level computation for traffic signal optimization through efficient weight processing and signal generation, substantially reducing vehicle waiting times and traffic congestion (**Figure** **4**a). Figure 4b illustrates how a single LVDS‐based system can efficiently control multiple traffic signals at an intersection, providing significant advantages in hardware complexity and power consumption compared to traffic control systems. The system processes traffic information through a three‐step process: first, it converts traffic information into electrical signals, then it generates device parameters, and finally, it determines optimal signal timing through our dual‐modulated synaptic device.

Given the deployment requirements of the target application, the implementation focused solely on the inference phase of the DDQN algorithm. In typical traffic control systems, reinforcement learning is conducted offline using software simulations, and the trained policy is then deployed across multiple physical intersections. This approach avoids the complexity of real‐time reward processing while offering significant benefits in energy efficiency, scalability, and hardware simplicity. The inference‐only design aligns well with real‐world deployment requirements, where robustness and low power consumption are critical (Figure S11, Supporting Information).

The device characterization results presented in Figure 4c demonstrate how our device combines two distinct weights ‒ “Advantage” weights (*W_A_*) and “Value” weights (*W_V_*) ‒ to generate final Q‐values (*W_Q_* = *W_A_* + *W_V_*) by translating neural network weights into physical device parameters. Processing these weight combinations yields an 80 × 80 normalized matrix that represents the combined DDQN layer weights. As shown in Figure 4d, Value‐layer weights ranging from −0.3 to 0.3 were mapped to corresponding light intensity levels ranging from 10 to 80 µW across 80 discrete steps. Figure 4e shows how Advantage‐layer weights ranging between −0.6 and 0.6 were mapped to gate voltages ranging from −3.5 to −7 V. Figure 4f illustrates the array‐scale implementation, which enables parallel weight processing by applying different gate voltages to individual transistors while maintaining a uniform light intensity within each word line. The system determines optimal control signals by measuring bit line currents, with the maximum current indicating the most favorable action according to Kirchhoff's law.

We evaluated our device performance using Simulation of Urban Mobility (SUMO),^[^ ^43^ ^]^ a specialized traffic simulation tool. As shown in Figure 4g, the system exhibits a dynamic response to changing traffic conditions. For example, while monitoring East‐West traffic flow, the system maintains a stop signal when detecting a single waiting vehicle (case ①, shown by the maximum current in Q1), but it switches to a proceed signal when four vehicles are in the queue (case ②, indicated by the maximum current in Q2). Figure S12 (Supporting Information) shows the complete traffic volume data and signal states for all five intersections, demonstrating the comprehensive control capabilities of our system. Through 100 repeated simulations, our device‐based system exhibited high consistency with software simulation: the average maximum wait times were 3.58 ± 0.21 s for our device vs 3.39 ± 0.22 s for software (Figure 4h), and the average vehicle throughput was 1.86 ± 0.07 for our device vs 1.92 ± 0.04 vehicles for the software (Figure 4i). To ensure consistency between simulation and hardware characteristics, all weight values used in the simulation were derived from experimentally measured, positive conductance states of the LVDS devices. This allowed us to model realistic Q‐value computations under the constraints of the actual device physics (Figure S13, Supporting Information). These results confirm that our device demonstrates reliability for practical applications while maintaining the inherent advantages of reduced power consumption (Figure S14, Supporting Information) and computational overhead.

While our current implementation successfully demonstrates device‐level inference using DDQN for traffic signal optimization, it is limited to the forward pass and does not support reward‐driven weight updates within the device. Realizing full on‐device learning will require hybrid analog‐digital interfaces capable of translating environmental feedback into weight modulation, and scaling to larger systems may pose challenges related to analog variability and stability. Nonetheless, the direct correspondence between our device operation and DDQN computation positions our approach as a scalable solution for more complex reinforcement learning tasks in the future.

## 3 Conclusion

By leveraging the device‐level summation capabilities of the LVDS transistor, we successfully implemented a DDQN‐based traffic signal optimization, significantly reducing system complexity while improving computational efficiency. This device‐level summation is accomplished through the independent optical and electrical memory functions enabled by the OLA ligand attached to the InAs QDs. Specifically, the LVDS transistor performs a linear summation of voltage and light inputs, which is utilized for DDQN computations. We evaluated the DDQN algorithm by applying it to traffic signal optimization using the LVDS transistor, and the results closely matched the ideal values, confirming the excellent performance of the LVDS transistor. Unlike previously reported synaptic transistors, the LVDS transistor can perform summation within a single device, maximizing computational efficiency and considerably reducing system complexity. Since this functionality is not limited to a specific algorithm but is applicable across various AI models, we anticipate that, when combined with appropriate optical signal processing technologies such as waveguides, it could enable efficient and practical implementations in on‐device AI systems.

## Conflict of Interest

The authors declare no conflict of interest.

## Author Contributions

D.G.R., S.C., and S.I. contributed equally to this work. J.H.C., S.J., and H.J. supervised and initiated the project. D.G.R. and S.I. conceived the project idea. S.C. and Y.Y. performed the device fabrication and electrical characterization. S.C. and M.K. conducted the material synthesis and physical analyses. S.K. and J.W.K. conducted the UPS analysis. S.I. performed and analyzed the simulation. D.G.R., S.I., and M.K. wrote the original draft of the manuscript. All the authors read and commented on the manuscript.

## Supporting information



Supporting Information

## Data Availability

The data that support the findings of this study are available from the corresponding author upon reasonable request.
