# Supplementary material for: Analog Signal Summation for Reinforcement Learning via Simultaneous Light–Voltage Modulation in a Synaptic Device
Source: Adv Sci (Weinh). 2025 Dec 12;13(10):e21293. doi: 10.1002/advs.202521293 (PMC12915106; doi:10.1002/advs.202521293)
Supplement: Supplementary file 1 — Supporting Information [file ADVS-13-e21293-s001.docx]

Supporting Information for

**Analog Signal Summation for Reinforcement Learning via Simultaneous Light–Voltage Modulation in a Synaptic Device**

*Dong Gue Roe^†^, Sungjoon Cheon^†^, Seongil Im^†^, Sinil Choi, Meeree Kim, Subeen Kim, Youngjae Yoo, Jeong Won Kim, Hyunsu Ju*, Sohee Jeong*, and Jeong Ho Cho**

**METHODS**

***Materials***. Indium(I) chloride (InCl; anhydrous, 99.99%, Alfa Aesar), trioctylphosphine (TOP; 97%, Aldrich), tris(dimethylamino)arsine (As(NMe_2_)_3_; 99%, Strem), chlorobenzene (anhydrous, Aldrich), hexane (anhydrous, Aldrich), and n-butyl alcohol (anhydrous, ≥99.5%, Aldrich) were used as supplied, whereas OLA (70%, Aldrich) was distilled under vacuum at 140 °C before use.

***InAs QD synthesis***. The InAs QDs were prepared using a previously reported method^[1]^ with slight modifications. In a glove box, 1.5 mmol of InCl was mixed with 7.2 mL of OLA and 0.7 mmol of TOP in a 100 mL three-neck flask and degassed under vacuum at 100 °C for 1 h. After switching to an N_2_ atmosphere, the mixture was gradually heated to 280 °C and maintained at this temperature for 10 min to ensure stability. In a separate vial (10 mL) inside the glove box, 0.8 mmol of As(NMe_2_)_3_ was mixed with 2 mL of OLA, and the mixture was heated to 50 °C for 5 min until bubbling stopped. Next, 1.3 mL of this arsenic precursor solution was rapidly injected into an indium precursor pot, and the reaction mixture was maintained at 280 °C for 28 min. Subsequently, the flask was cooled to room temperature, and 8 mL of the crude solution was mixed with 2 mL of hexane, followed by 30 mL of butanol. The mixture was centrifuged at 6000 rpm for 5 min to collect precipitates. This step was repeated twice. The final precipitate was collected and redispersed in 2 mL of chlorobenzene.

***Ligand exchange***. Ligand exchange between oleylamine (OLA) and 3-mercapto-1,2-propanediol (MPD) was performed according to a modified version of a previously reported method^[2]^. A solution of 7 mL CQDs in octane (10 mg mL⁻¹) was mixed with 7 mL of ligand solution prepared by dissolving 75 μL of butylamine and 150 μL of 1-thioglycerol in DMF. The mixture was vortexed for 1 min to initiate ligand exchange. After phase separation, the DMF phase containing ligand-exchanged CQDs was collected and washed twice with fresh n-octane. CQDs were then precipitated by the addition of toluene and centrifuged at 6000 rpm for 4 min. The final pellet was dried under vacuum, redispersed in DMF, and filtered prior to spin-coating. All procedures were performed inside a nitrogen-filled glove box.

***LVDS transistor fabrication***. The LVDS transistor was fabricated on a SiO₂/Si substrate. Before fabrication, the substrates were sequentially cleaned via sonication in acetone, isopropyl alcohol, and deionized (DI) water. The prepared InAs QD solutions were spin-coated onto the substrate at 3000 rpm for 45 s. IGZO was then deposited by RF sputtering and patterned using conventional photolithography and wet etching with AZ 5214E (AZ Electronic Materials) and an ITO etchant (DI water:LCE-12 ratio = 3:1, Cyantek). Finally, aluminum source and drain electrodes (40 nm) were selectively deposited via thermal evaporation through a shadow mask.

***Measurement***. The electrical properties of all the devices were measured using Keithley, 4200A-SCS. The TEM images of InAs nanocrystals were obtained using a JEM-ARM200CF (JEOL) at 200 kV (**Figure 2f**) and JEM-F200 (JEOL) at 50 kV (**Figure S1**). The UV–Vis-infrared absorbance spectra of the InAs nanocrystals were acquired using a UV–Vis/NIR spectrophotometer (Shimadzu UV-3600). The FT-IR spectra were collected using a Thermo Fisher Nicolet iS50. The FT-IR samples were prepared by spin-coating on a Si/Cr/Au substrate. The ^1^H NMR spectrum was recorded using a 500 MHz NMR (Bruker) with 32 scans at the Chiral Material Core Facility Center of Sungkyunkwan University. The samples were prepared in deuterated toluene (toluene-*d_8_*). UPS measurements were conducted using a He–I discharge lamp (*ħω* = 21.2 eV) and analyzed using a hemispherical electron analyzer (VG-Scienta) under ultrahigh vacuum conditions (<10^−8^ Torr). The wavelength of the light used as the synaptic input was 1060 nm. The light power indicated throughout the manuscript refers to the effective power, which was calculated using the following formula: Effective light power = (Incident light power / Laser spot area) × Channel width × Channel length.

***Device-Level Encoding of DDQN Weights.*** To enable device-level implementation of DDQN, all trained weights were first linearly shifted into the positive domain to match the unipolar conductance characteristics of the LVDS transistor. These weights were then quantized into 80 discrete levels and mapped to physical stimuli: Value weights were assigned to light intensities (10–80 μW), and Advantage weights to gate voltages (–3.5 to –7.0 V). This mapping was chosen based on the device’s stronger optical response for shared Value modulation and finer electrical tunability for action-specific Advantage control. Importantly, the mapping process preserved the relative order of weights rather than their absolute values, ensuring that Q-value ranking—and therefore DDQN decision logic—remained intact. To maintain realism while supporting system-scale simulation, experimentally measured 8×8 conductance data were interpolated into an 80×80 array, allowing reliable performance evaluation under physically grounded constraints.

[1] M. Ginterseder, D. Franke, C. F. Perkinson, L. Wang, E. C. Hansen, M. G. Bawendi, *Journal of the American Chemical Society* **2020**, 142, 4088.

[2] J. Leemans, V. Pejović, E. Georgitzikis, M. Minjauw, A. B. Siddik, Y. H. Deng, Y. Kuang, G. Roelkens, C. Detavernier, I. Lieberman, *Advanced Science* **2022**, 9, 2200844.


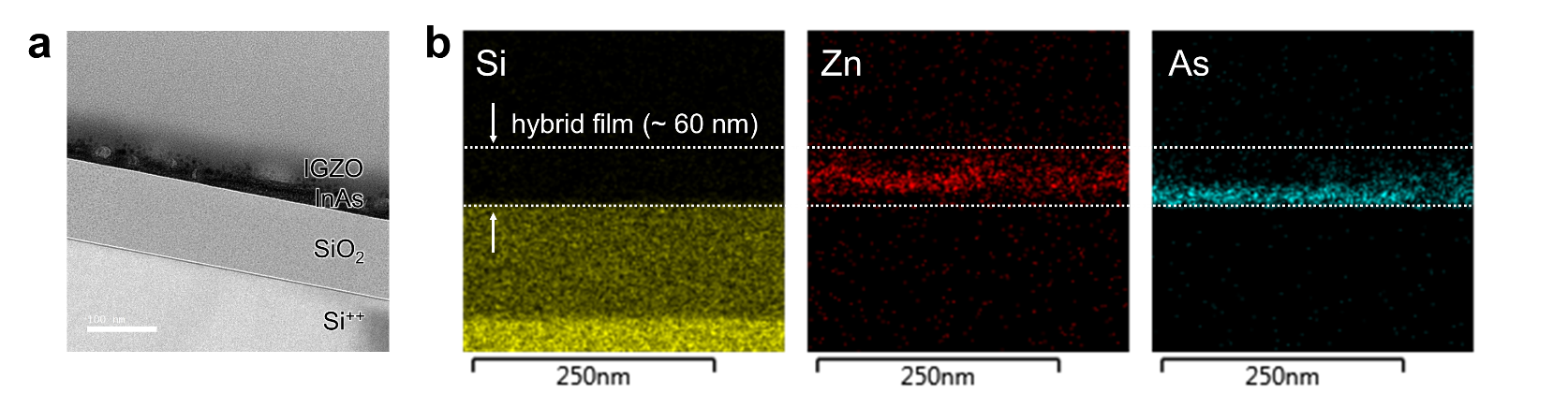


**Figure S1.** (a) Cross-sectional TEM image of the InAs QD and IGZO hybrid layer deposited on a SiO₂/Si⁺⁺ substrate. (b) TEM-EDS elemental mapping of the hybrid layer.


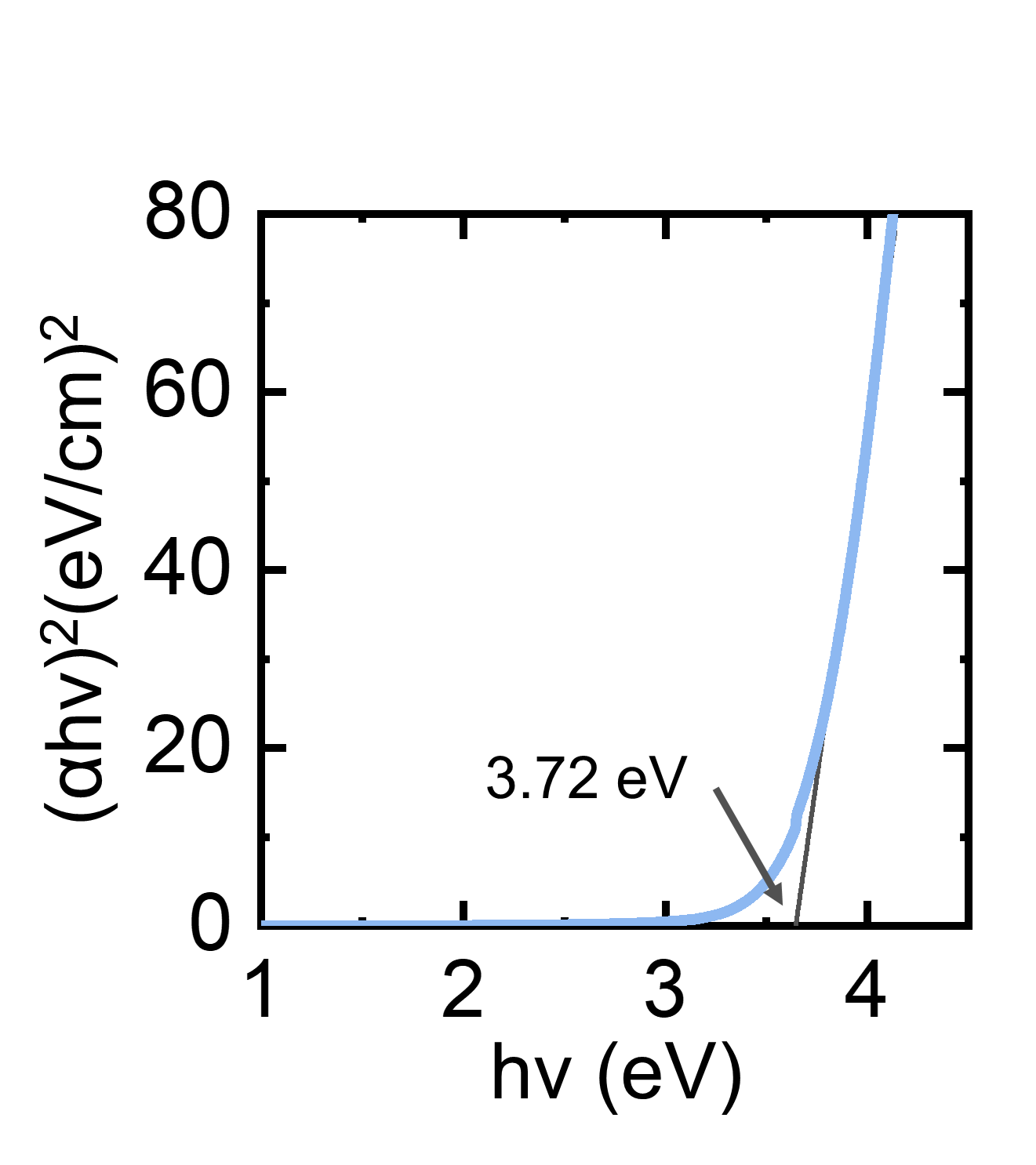


**Figure S2.** Tauc plot of IGZO.

**
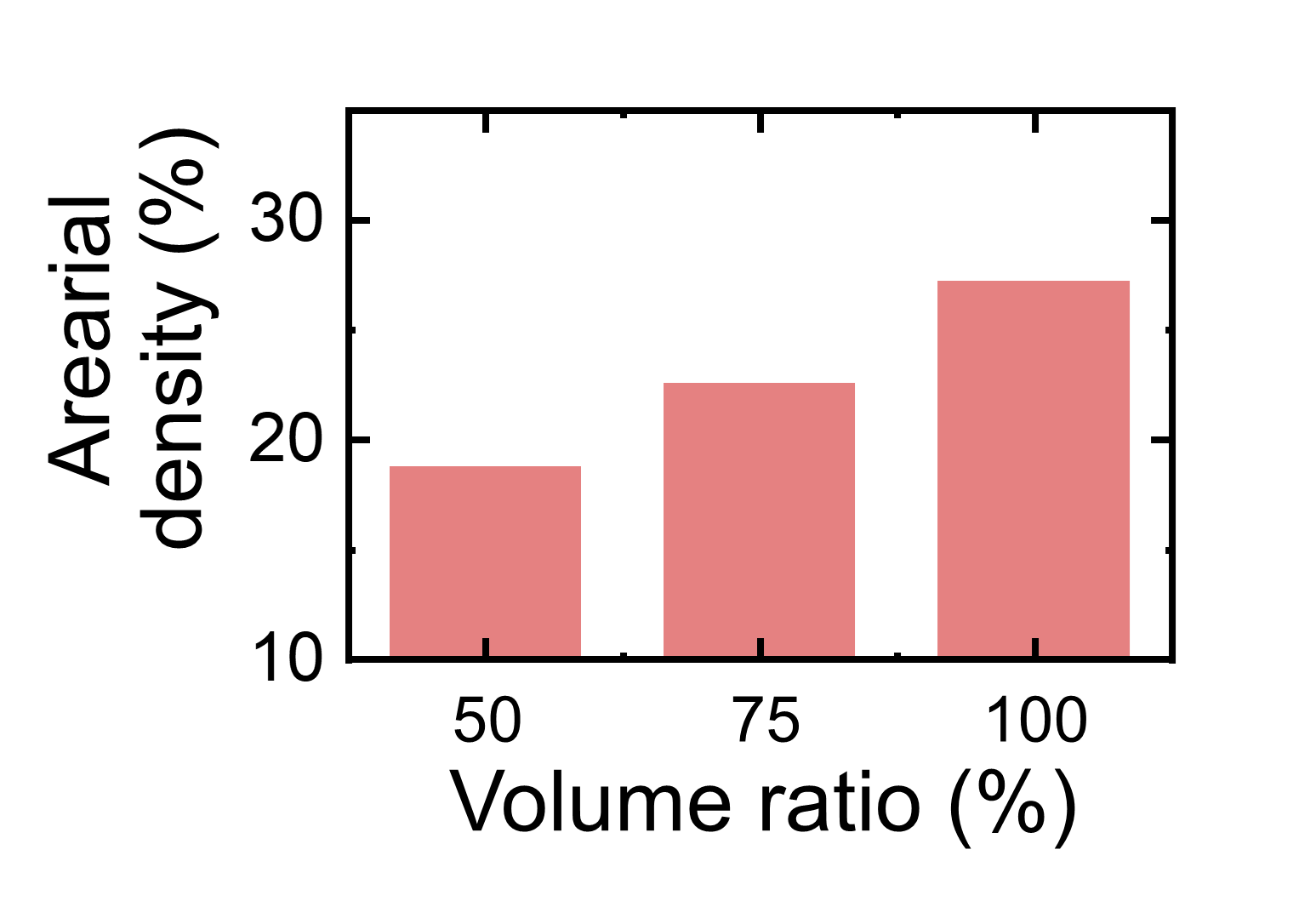
**

**Figure S3.** Aerial density of spin-coated InAs QDs at varying volume ratios.


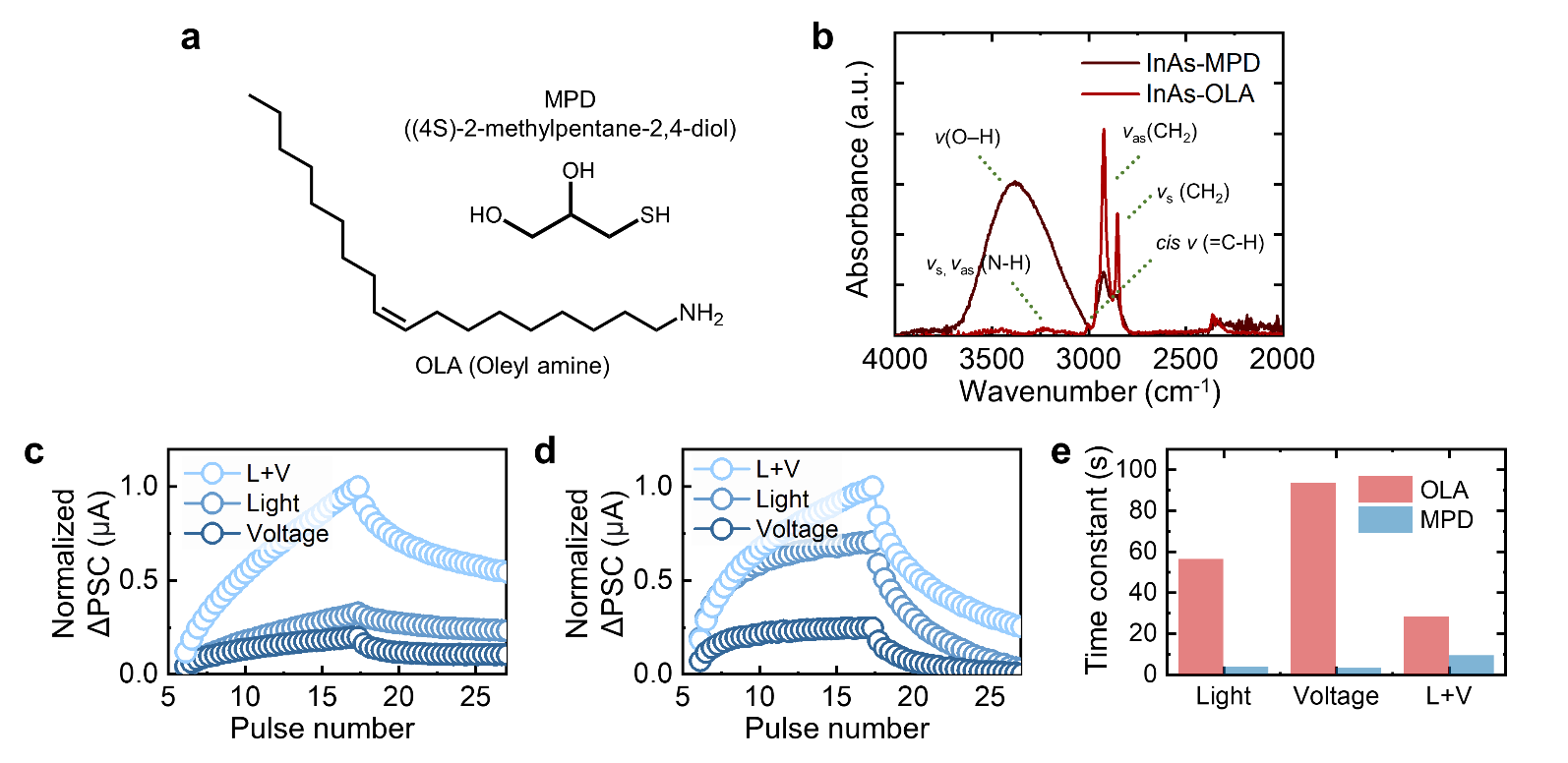


**Figure S4.** Characterization of the LVDS transistor fabricated with MPD attached InAs QDs. (a) Molecular structure of the OLA and MPD. (b) FT-IR spectrum of OLA and MPD attached InAs QDs. (c) Retention characteristics of the LVDS transistor based on OLA attached InAs QDs and (d) MPD attached InAs QDs under potentiation condition (30 pulses, light: 80 μW, voltage: -8 V). (e) Comparison of retention time constants between devices based on OLA- and MPD-attached InAs QDs.

**
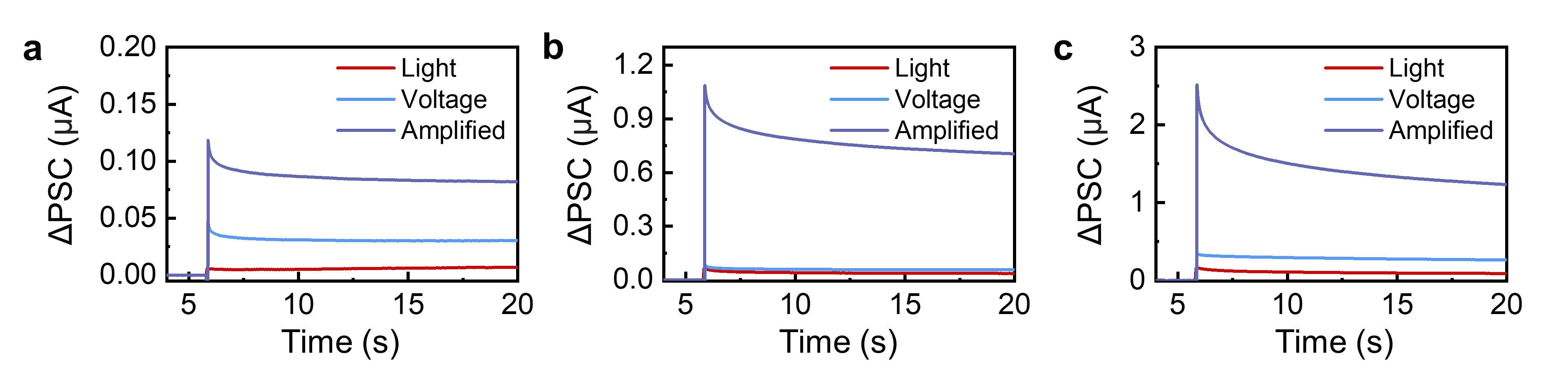
**

**Figure S5.** PSC changes under various input conditions for InAs QD solutions with (a) a 50% volume ratio, (b) a 70% volume ratio, and (c) a 100% volume ratio.


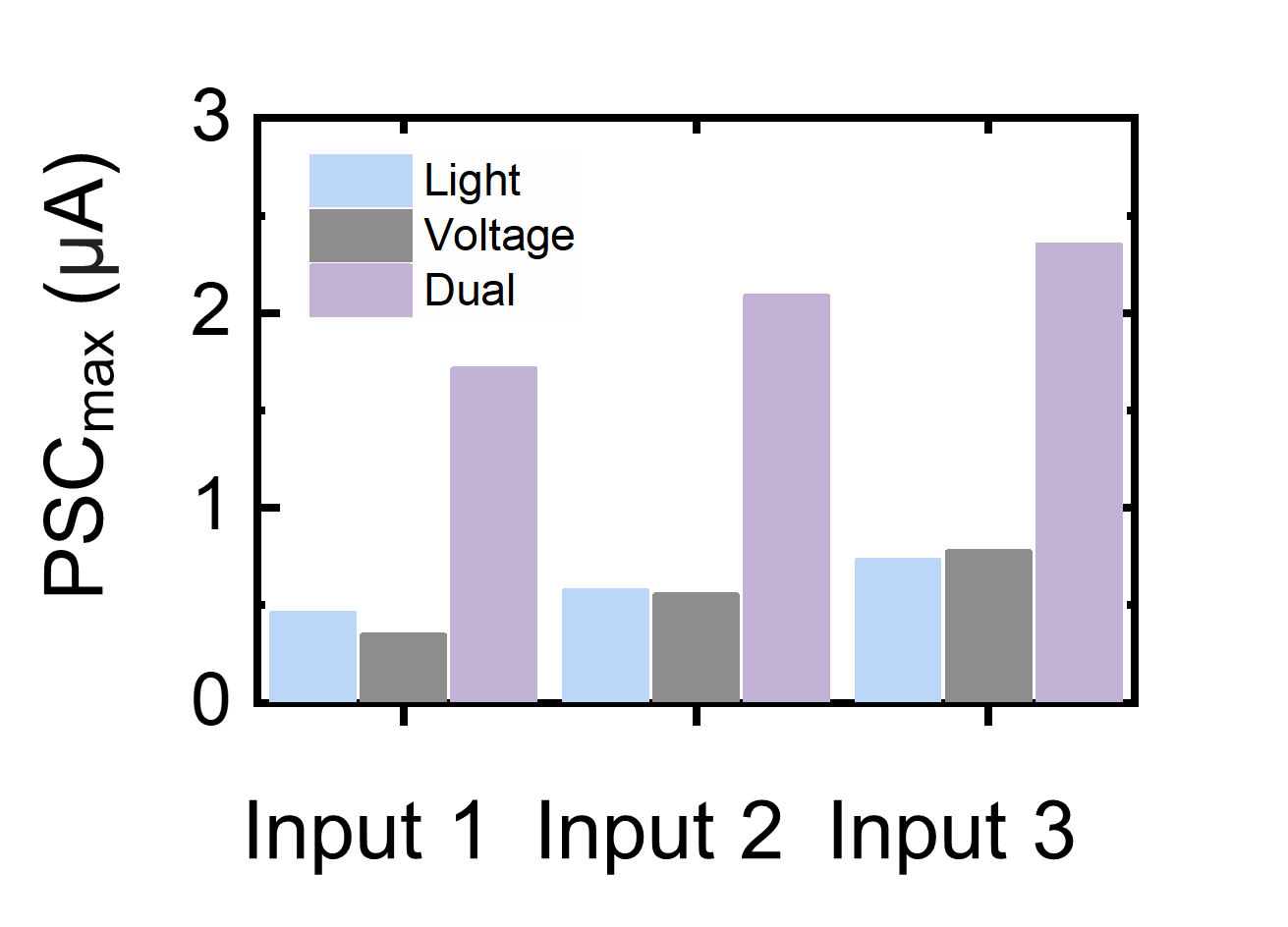


**Figure S6.** PSC comparison between single inputs and simultaneous light and voltage inputs.


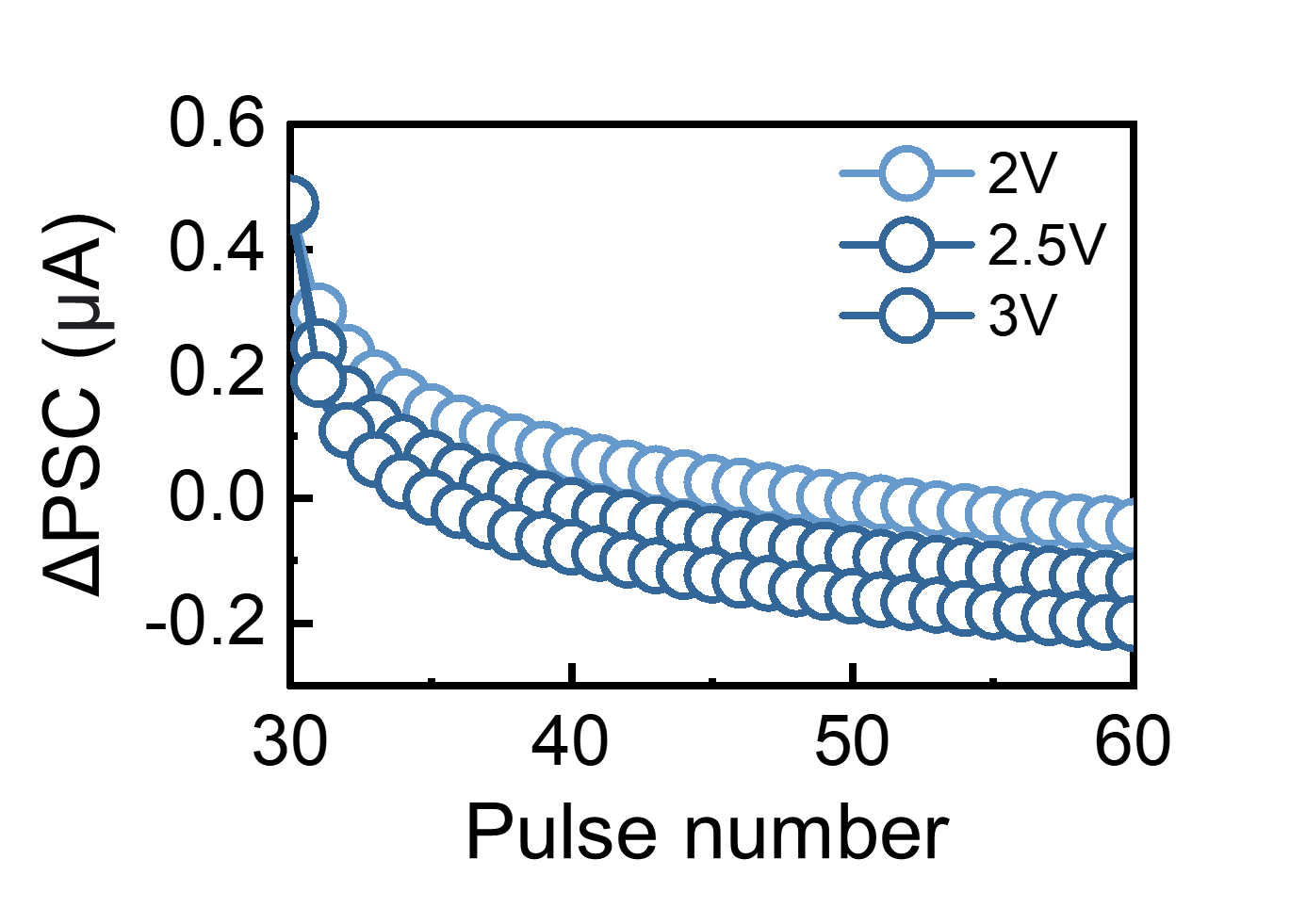


**Figure S7.** LTD characteristics under different input voltage amplitudes.


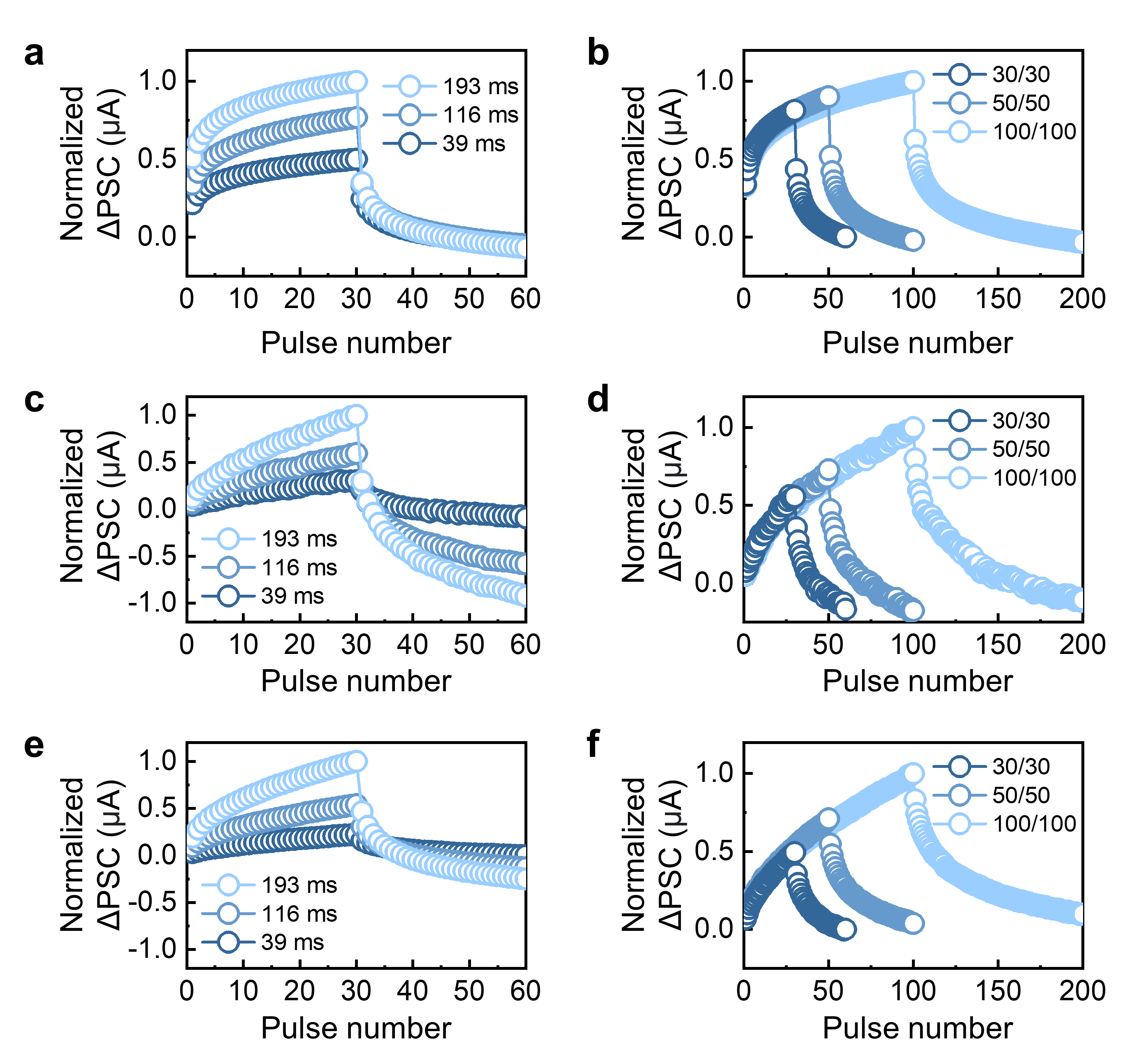


**Figure S8.** Voltage-induced LTP/D curves under varying (a) pulse width and (b) pulse number. Light-induced LTP/D curves under varying (c) pulse width and (d) pulse number. LTP/D curves induced by simultaneous light and voltage pulses under varying (e) pulse widths and (f) pulse numbers.


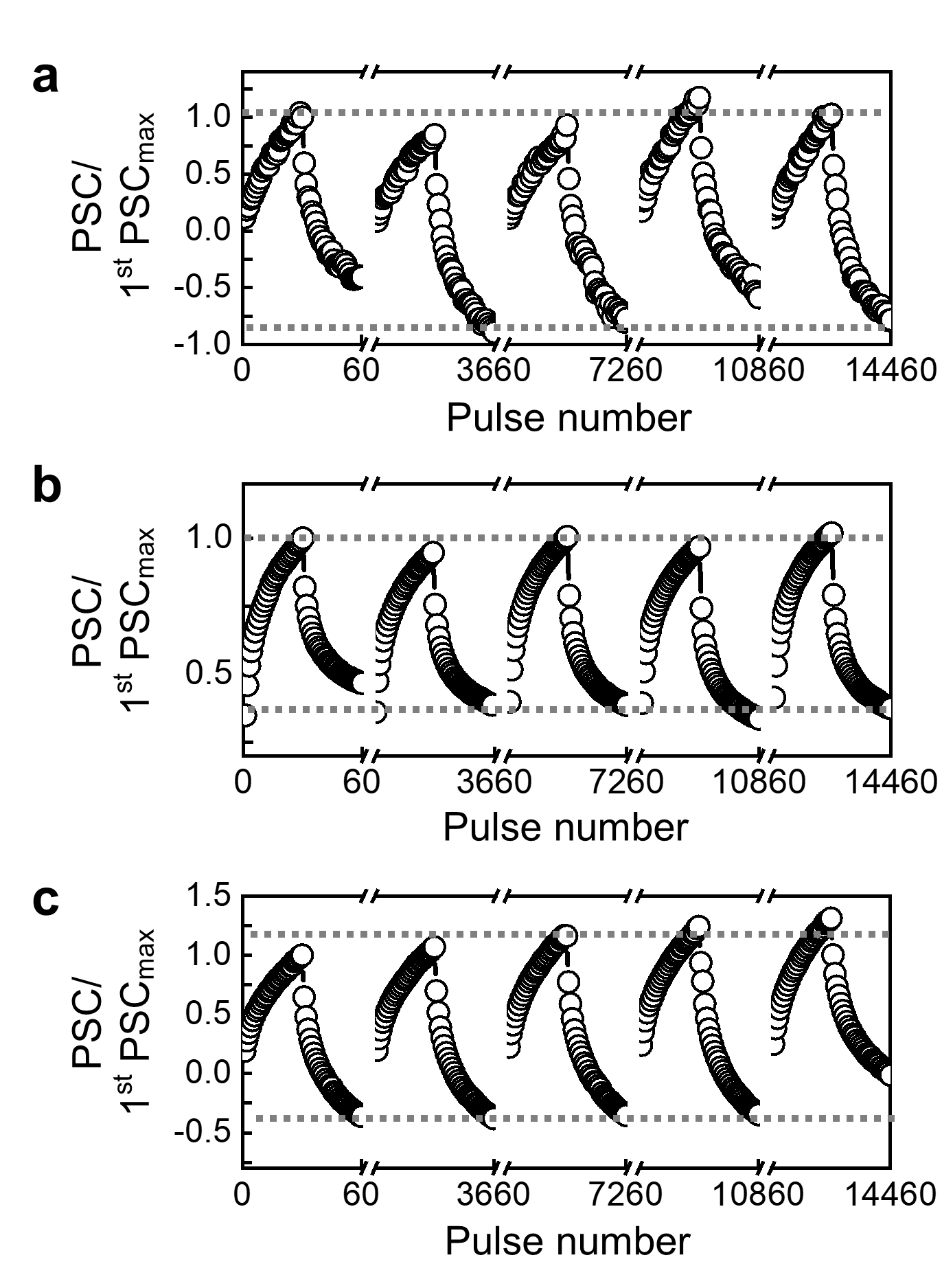


**Figure S9.** Device stability under consecutive pulse conditions: (a) Potentiation: -80 µW, depression: +1 V; (b) Potentiation: -7 V, depression: +2 V; (c) Potentiation: -8 V and -80 µW, depression: +3 V.


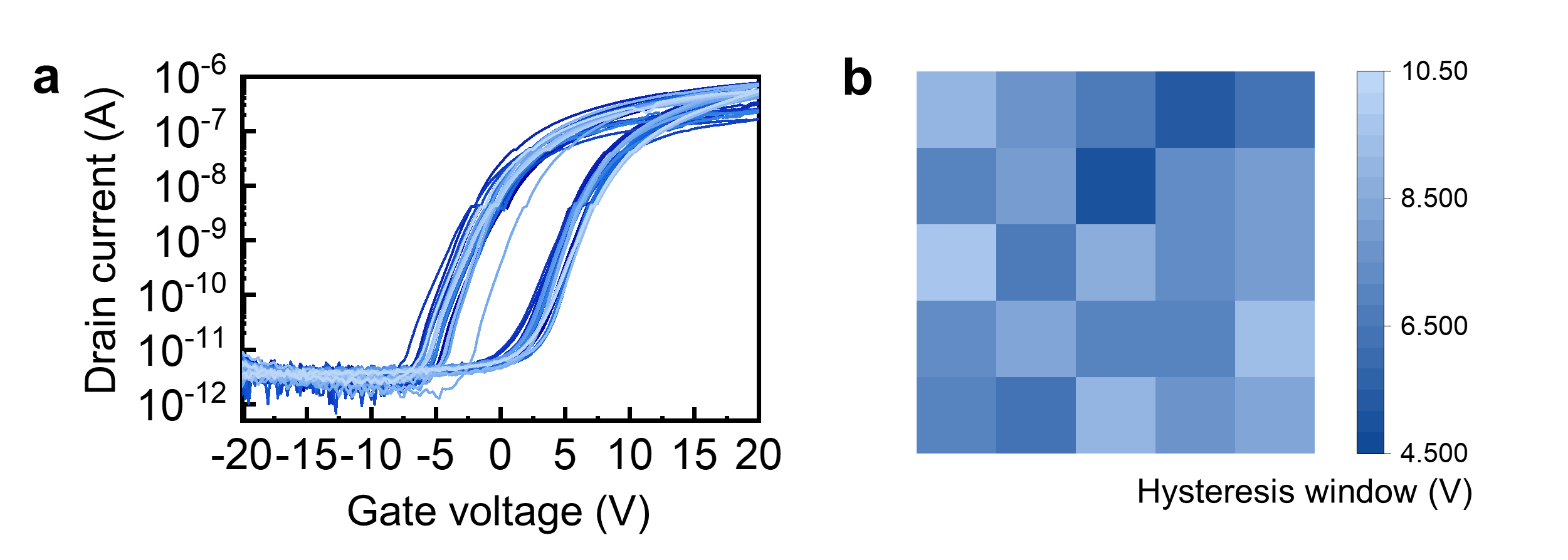


**Figure S10.** (a) Transfer curves of the 25 LVDS transistors (*V*_DS_ = 1 V) and (b) their respective calculated hysteresis windows.


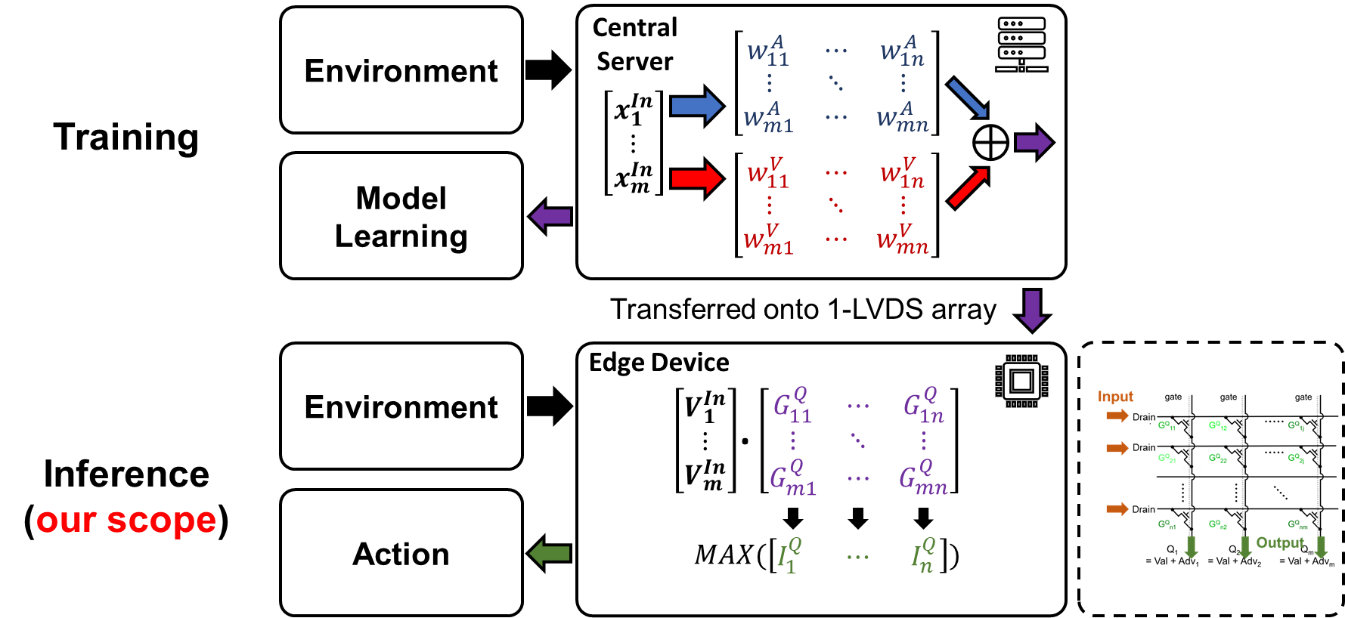


**Figure S11. System architecture distinguishing training and inference phases in DDQN implementation.** Training occurs on central servers using conventional computing resources, while the trained weights are transferred to LVDS device arrays for real-time inference at edge deployment sites. Our implementation focuses on the inference phase, enabling energy-efficient decision-making.

**
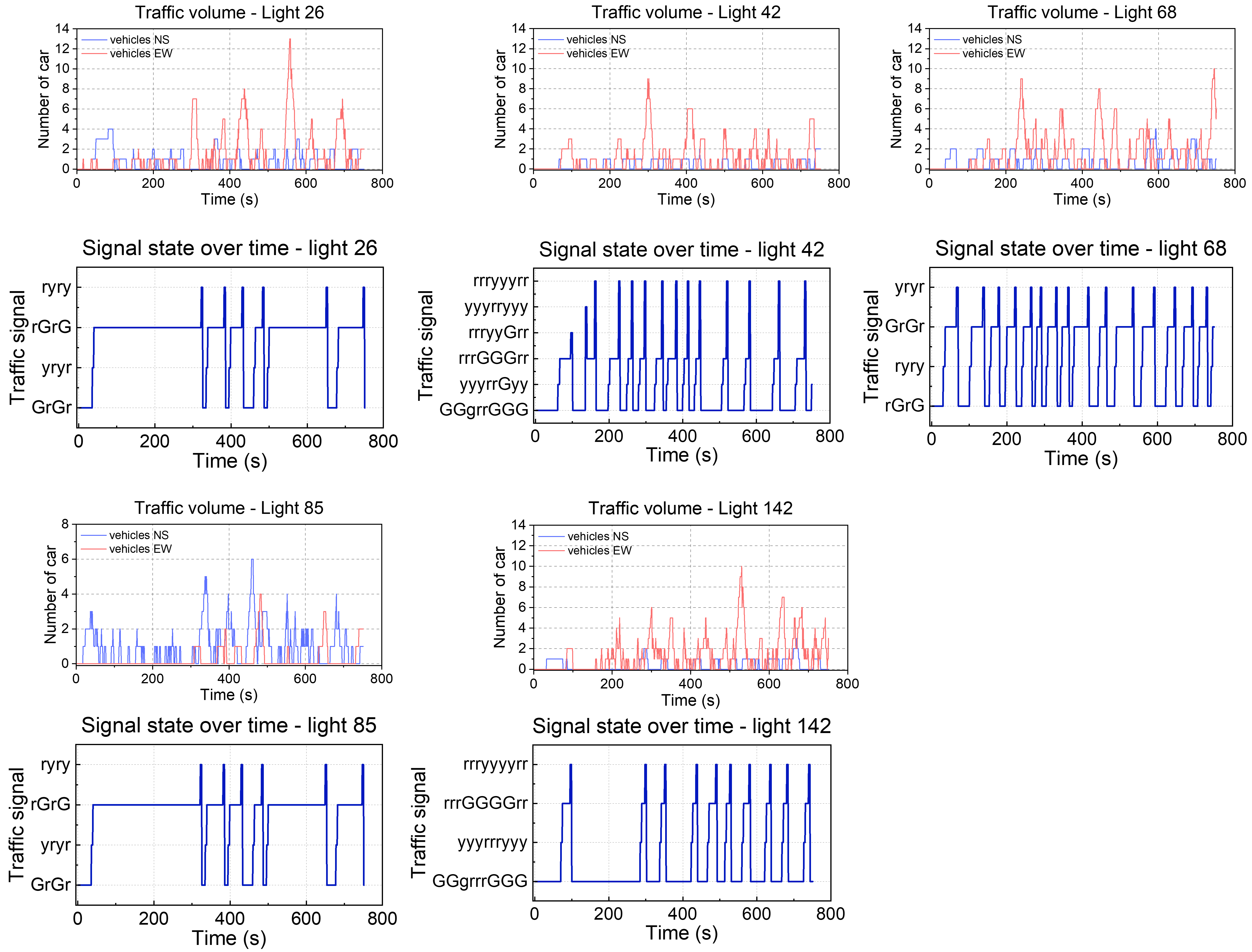
**

**Figure S12.** Comprehensive traffic flow and signal states across five intersections under DDQN-based control.


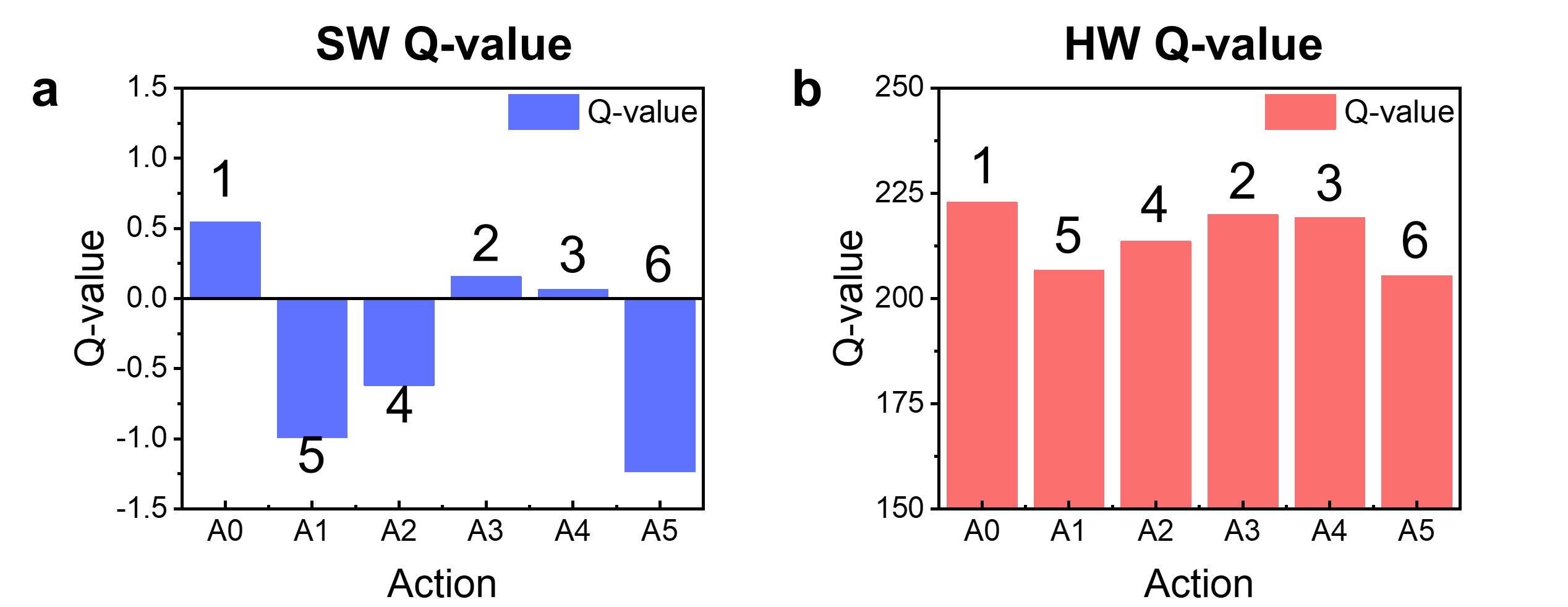


**Figure S13. Q-value comparison between software and hardware implementations for DDQN-based traffic control.**

Q-values for six traffic control actions computed using (a) original software DDQN and (b) hardware device implementation with dual light-voltage modulation for an example traffic scenario. Numbers indicate action rankings (1=highest priority). The hardware implementation shows positive-shifted values due to unipolar device constraints but preserves identical action rankings (A0 > A3 > A4 > A2 > A1 > A5), demonstrating that the dual-modulation mechanism successfully maintains DDQN decision-making logic while operating within hardware limitations.


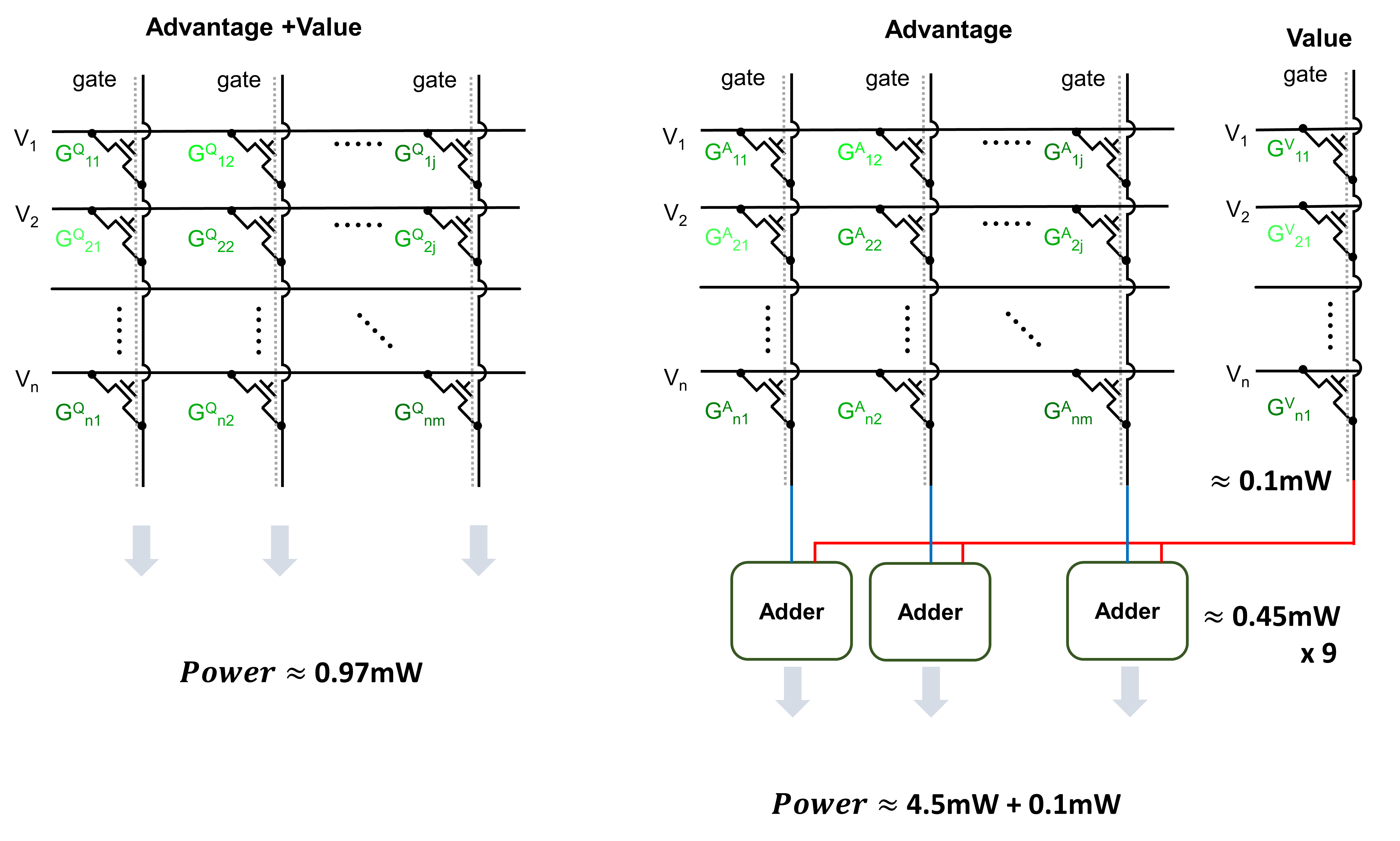


**Figure S14.** Comparison of power consumption between the light–voltage dual-modulating array and conventional methods.

Power consumption analysis revealed that the light–voltage dual-modulating array computes both Advantage (voltage) and Value (light) within the array, accounting for only the intrinsic transistor power dissipation. In contrast, conventional methods compute Advantage (voltage) and Value (voltage) separately, requiring a bit-wise adder for each Action in the DDQN. Thus, the light–voltage dual-modulating array consumes 0.97 mW of power, whereas the conventional method consumes 4.6 mW (a typical low-powered adder consumes ~0.45 mW).
